# Alphataxin, an Orally Available Small Molecule, Decreases LDL Levels in Mice as a Surrogate for the LDL-Lowering Activity of Alpha-1 Antitrypsin in Humans

**DOI:** 10.3389/fphar.2021.695971

**Published:** 2021-06-09

**Authors:** Cynthia L. Bristow, Ronald Winston

**Affiliations:** ^1^ Alpha-1 Biologics, Long Island High Technology Incubator, Stony Brook University, Stony Brook, NY, United States; ^2^ Institute for Human Genetics and Biochemistry, Vesenaz, Switzerland

**Keywords:** α1-proteinase inhibitor, elastase, alphataxin, cholesterol, lactams, ezetimibe

## Abstract

The abundant blood protein α1-proteinase inhibitor (α1PI, Αlpha-1, α1-antitrypsin, *SerpinA1*) is known to bind to the active site of granule-associated human leukocyte elastase (HLE-G). Less well known is that binding of α1PI to cell surface HLE (HLE-CS) induces lymphocyte locomotion mediated by members of the low density lipoprotein receptor family (LDL-RFMs) thereby facilitating low density lipoprotein (LDL) clearance. LDL and α1PI were previously shown to be in negative feedback regulation during transport and clearance of lipoproteins. Further examination herein of the influence of α1PI in lipoprotein regulation using data from a small randomized, double-blind clinical trial shows that treatment of HIV-1-infected individuals with α1PI plasma products lowered apolipoprotein and lipoprotein levels including LDL. Although promising, plasma-purified α1PI is limited in quantity and not a feasible treatment for the vast number of people who need treatment for lowering LDL levels. We sought to develop orally available small molecules to act as surrogates for α1PI. Small molecule β-lactams are highly characterized for their binding to the active site of HLE-G including crystallographic studies at 1.84 Å. Using high throughput screening (HLE-G inhibition, HLE-CS-induced cellular locomotion), we show here that a panel of β-lactams, including the LDL-lowering drug ezetimibe, have the capacity to act as surrogates for α1PI by binding to HLE-G and HLE-CS. Because β-lactams are antibiotics that also have the capacity to promote evolution of antibiotic resistant bacteria, we modified the β-lactam Alphataxin to prevent antibiotic activity. We demonstrate using the diet-induced obesity (DIO) mouse model that Alphataxin, a penam, is as effective in lowering LDL levels as FDA-approved ezetimibe, a monobactam. Non-antibiotic β-lactams provide a promising new therapeutic class of small molecules for lowering LDL levels.

## Introduction

The abundant blood protein α1-proteinase inhibitor (α1PI, Αlpha-1, α1-antitrypsin, *SerpinA1*) binds to the active site of granule-associated human leukocyte elastase (HLE-G) forming a covalent-like complex that inactivates both HLE-G and α1PI (Beatty et al., 1980; Herve and Ghelis, 1991). In addition, α1PI binds to cell surface human leukocyte elastase (HLE-CS) at the leading edge of a migrating T lymphocyte and stimulates aggregation of functionally related receptors such as T cell antigen receptors (TCR), CD4, and CD184 (CXCR4) (Fuks et al., 1983; Bristow and Flood, 1993; Bangalore and Travis, 1994; Bristow et al., 2003; Janciauskiene and Welte, 2016). The receptor aggregate subsequently associates with members of the low density lipoprotein receptor family (LDL-RFMs) at the trailing edge of the migrating cell resulting in internalization (endocytosis) of the aggregated receptors and their cargo including CD4-coupled HIV, TCR-coupled antigens, and nutrients such as low density lipoprotein (LDL) (Bristow et al., 1991; Bristow et al., 2003; Bristow et al., 2013). Endocytosis at the trailing edge of the migrating cell stimulates NF-κB phosphorylation, and release of the trailing edge of the cell from the tissue matrix, a situation that propels the cell forward (Bristow et al., 2008; Bristow and Winston, 2021). This process, in common with many other cell surface proteinase/proteinase inhibitor pairs, is constitutive and provides a physical mechanism for CD4^+^ T lymphocytes to sample the environment for nutrients and toxins, induce nuclear signaling, and promote cellular locomotion (Chu et al., 1994; Elia et al., 2007; van den Bos et al., 2020).

The participation of LDL-RFMs during T cell locomotion stimulated by α1PI suggested that α1PI might participate in the regulation of LDL levels. We previously demonstrated that α1PI and LDL are in negative feedback regulation (Bristow et al., 2013). Within the endogenous lipoprotein transport pathway (transport through the liver), LDL is transported through blood and lymph *via* CD4^+^ T lymphocytes (Ho et al., 1976b; Ho et al., 1976a; Xie et al., 1999; Borrell-Pagès et al., 2011; Randolph and Miller, 2014). Within the exogenous lipoprotein transport pathway (transport from the intestines), fatty acids are absorbed from the intestinal lumen into intestinal cells (enterocytes) where fatty acids are processed to form chylomicrons that incorporate apolipoprotein B-48 (apoB48) in the outer coat with triglycerides (Feingold, 2021). These chylomicrons are secreted through the lamina propria into intestinal lymphatics (lacteals) (Tso and Balint, 1986; Hokkanen et al., 2019). Intestinal lymphatics, which differ from plasma lymphatics, transport chylomicrons through mesenteric lymph nodes into the thoracic duct and to the bloodstream thereby bypassing the liver (Randolph and Miller, 2014). The mechanisms of chylomicron transport through the intestinal lymphatics are not well understood.

Whereas apoB48 does not bind to LDL-RFMs, enterocytes also express α1PI which does bind to LDL-RFMs (Tso and Balint, 1986; Molmenti et al., 1993; Xie et al., 1999). We hypothesized that chylomicron internalization by intestinal dendritic cells, macrophages, or lymphocytes is induced in the presence of α1PI. Here, we show that α1PI treatment of HIV-1-infected individuals decreased circulating levels of apoB48 (48 kDa), but not circulating levels of apoB100 (100 kDa), both of which are synthesized from the same gene (Farese et al., 1996; Fisher, 2012). ApoB100 does bind to LDL-RFMs and incorporates into LDL and very low density lipoprotein (VLDL) in the liver (Feingold, 2021).

Elevated LDL is a risk factor for cardiovascular disease, the leading cause of premature death, and affects more than 30% of the global population (World Health Organization, 2017). The first line class of drugs for lowering LDL levels that are delivered orally include statins which are HMG-CoA reductase inhibitors that act to diminish cholesterol synthesis in the liver (Shepherd et al., 2003). A second class of drug is ezetimibe which inhibits absorption of cholesterol in the small intestines by enterocytes (Chow et al., 2009). In addition, PCSK9 inhibitors are monoclonal antibodies that are delivered by injection and block receptors for LDL (Natarajan and Kathiresan, 2016; Nissen et al., 2016). Each of these classes of drugs can be very effective, yet can have serious adverse side effects in some individuals. In a randomized clinical trial, 43% of those treated with statins had intolerable muscle symptoms (NCT01984424), although similar symptoms were reported by 27% of those in the placebo arm (Nissen et al., 2016). Ezetimibe and PCSK9 inhibitors also caused muscle symptoms in 28 and 21% of individuals, respectively; ezetimibe was significantly less effective in lowering LDL levels than PCSK9 inhibitors (*p* < 0.001) (Nissen et al., 2016). Thus, identifying α1PI as a component of a previously unrecognized pathway involved in LDL regulation introduces the potential for novel treatments particularly in those who are unable to tolerate statins, ezetimibe, or PCSK9 inhibitors.

Using α1PI for treatment of the large percentage of the population exhibiting elevated LDL is not feasible because α1PI is purified from plasma, is limited in quantity, and requires frequent infusion. We sought to develop an orally available small molecule to act as a surrogate for α1PI to complex with HLE-CS thereby inducing cellular locomotion and increasing transport and clearance of LDL *via* LDL-RFMs. Small molecule β-lactams are highly characterized including their binding to the active site of HLE-G in crystallographic studies at 1.84 Å (Navia et al., 1987; Bonney et al., 1989; Doherty et al., 1993). These data suggested that β-lactams might be useful candidates to act as surrogates for α1PI.

To determine whether β-lactams might be useful for stimulating cellular locomotion and transporting lipoproteins, literature was reviewed that described antibiotic efficacy some of which had examined the effect of antibiotics on lymphocytes *in vitro*. Cephalosporins were found to suppress lymphocyte responses to pokeweed mitogen, phytohemagglutinin and concanavalin A, all of which stimulate subsets of T lymphocytes (Chaperon and Sanders, 1978). Some cephems showed greater suppression of bone marrow progenitor cells *in vitro* than penams (Charak et al., 1991). Some studies had examined the influence of β-lactam treatment on blood cell counts *in vivo*, and a very few publications had also examined effects on cholesterol levels in uninfected control animals. A form of cefuroxime was examined in uninfected beagles and showed an increase in reticulocytes in treated animals as compared with untreated animals and a decrease in leukocytes during treatment in female animals; however, cholesterol levels were consistently lower in treated animals in comparison with untreated animals even though triglyceride levels were significantly greater in treated as compared with untreated animals suggesting the effect was associated with liver production of lipoproteins as opposed to fat absorption by enterocytes (Spurling et al., 1986). No abnormalities were detected in adrenals, aorta, urinary bladder, caecum, colon, duodenum, ileum, jejunum, lymph nodes, ovaries, pancreas, pituitary, testes, or uterus in that study. In other studies, cephalosporin, but not ampicillin, was shown to increase leukopenia in human patients with liver impairment, and several β-lactams including ezetimibe were shown to inhibit cholesterol absorption in humans (Singh et al., 1993; Burnett et al., 1994). Based on review of these studies, we decided to first examine *in vitro* interactions of β-lactams with HLE-G and HLE-CS using high throughput screening followed by *in vivo* testing in mice. Whereas in humans, there is one gene encoding α1PI (*SerpinA1*), in mice there are as many as five genes with different mouse species expressing 1–5 gene variants (Forsyth et al., 2003). In mice, the HLE-G homolog encoded by the *ELANE* gene has 74.9% amino acid sequence identity with the human counterpart and 100% alignment of the active site amino acids His, Asp, and Ser using the Constraint-based Multiple Alignment Tool (COBALT) suggesting that mice are suitable for *in vivo* testing of Alphataxin.

We identify herein a panel of β-lactams that bind to HLE-G and HLE-CS. Our lead candidate, Alphataxin, is a levorotary β-lactam that lacks effective antibacterial activity and differs functionally and physically from its dextrorotary antibacterial enantiomer. Enantiomers are discrete molecules requiring stand-alone FDA approval studies due to their physical and functional differences. We show in preclinical *in vivo* studies using the Jackson Laboratory diet-induced obesity mouse model of human metabolic syndrome and diabetes (DIO) that Alphataxin, a penam, effectively lowered LDL levels equivalently to ezetimibe, a monobactam.

## Materials and Methods

**Clinical trial NCT01731691**: This clinical trial was a double-blind, randomized study as previously described (Bristow et al., 2019). Written informed consent was received from 12 individuals, 8 HIV-1-infected individuals and 4 uninfected controls. Blood was collected weekly at the same time of day at baseline and for 8 subsequent weeks from uninfected, untreated controls and from HIV-1-infected individuals who were treated weekly with Prolastin-C (n = 3) or with placebo (n = 5). Inclusion criteria for HIV-1 infected subjects were: 1) active α1PI below 11 μM; 2) one year history with CD4^+^ lymphocytes at levels ranging between 200 and 600 cells/μl; 3) absence of symptoms suggestive of HIV-1 disease progression; 4) adequate suppression of virus (<1,000 HIV RNA/ml); and 5) history of compliance with antiretroviral medication. Inclusion criteria HIV-1 uninfected, untreated controls were the same as for HIV-1 infected subjects excluding HIV-related criteria. Grifols Biotherapeutics contributed a sufficient quantity of Prolastin-C (lot# 26NLK52) for administration of 8 weekly infusions at a dose of 120 mg/kg. The study protocol was approved by Copernicus Group Independent Institutional Review Board, Durham, NC. Drug delivery and blood collection were performed at ACRIA, New York, NY. No adverse effects were reported by any volunteers, and all volunteers remained in the study for the full period.

**Human apolipoproteins measurement**. Total cholesterol, high density lipoprotein (HDL), low density lipoprotein (LDL), triglycerides, apolipoprotein E (apoE), and apolipoprotein A1 (apoA1) measurements were performed by ICON Central Laboratory, Farmingdale, NY. Cholesterol, HDL, and triglycerides were measured using the Chol2, HDLC3, TRIGL methods on the Roche/Hitachi cobas c analyzer. LDL was calculated using the formula total cholesterol – HDL – (triglycerides/5). ApoE was quantitated using the Kamiya Biomedical Co. K-assay immunoturbidimetric method. ApoA1 was quantitated using the Siemens N antisera method by immunonephelometry on the BN II and BN ProSpec System. Total apoB and apoB48 measurements were contracted by ICON Central Laboratory to Nexelis (formerly Pacific Biomarkers). Total apoB was measured in serum by immunoturbidimetry using the Roche Modular P instrument and Goat polyclonal antibody specific for human apoB. ApoB48 wase measured by sandwich ELISA using a capture monoclonal antibody and detection using a biotin-conjugated polyclonal antibody followed by HRP-conjugated Streptavidin and addition of a chromogenic substrate using a SoftMax Pro ELISA plate reader. ApoB100 was calculated as total apoB minus apoB48. Cholesterol, triglycerides, HDL, LDL, apoE and apoA1 were measured by ICON Central Laboratory, Farmingdale, NY.

**Animal Studies**. Animal studies were performed by Jackson Laboratory (Bar Harbor, ME), an OLAW-Assured and AAALAC accredited institution. All research was reviewed and approved by the Institutional Animal Care and Use Committee (IACUC) prior to commencing. The study used 16–17 weeks old male DIO C57BL/6J mice that had been maintained on a 10% (control) or 60% high-fat diet. For drug comparison, mice on the 60% high-fat diet were randomly assigned to three groups with 12 mice per group: Vehicle Control, Zetia (ezetimibe, 10 mg/kg), and Alphataxin (5 mg/kg). Treatment was delivered by daily oral gavage at the same time of day for 12 weeks. Blood was collected by retro-orbital bleed every 3 weeks for measurement of total cholesterol, LDL, HDL, triglycerides, non-essential free fatty acids and glucose using the Beckman Coulter AU680 Chemistry System.

Food consumption was monitored twice weekly, and body weight was monitored weekly. As mice gained weight on the 60% high-fat diet during the study, dosing was adjusted accordingly. Mice were monitored for adverse effects (e.g., labored breathing, aberrant weight change). Three mice were lost during the study. Three mice were lost in the ezetimibe group, and one in the Alphataxin (5 mg/kg) group. The mice lost in the ezetimibe group were due to a severe bite wound and an unknown causes. The mouse lost in the Alphataxin group was due to an abnormal amount of gas in the stomach attributed to a gavaging error.

**Cells and Reagents**. Human U937 Clone 10 promonocytic cells (a generous gift of the Laboratory of Immunoregulation, NIAID, NIH) or murine F12.23 cells (a generous gift of Mark I. Greene, Univ. Penn.) were selected because they have CD4^+^ T lymphocyte characteristics (Bristow et al., 1991; Bristow et al., 2008). Cells were cultured using RPMI-1640 containing 10% FBS. Prior to use, cells were cultured overnight in AIM-V serum-free medium (Thermo Fisher Scientific).

Human leukocyte elastase (HLE-G, EC 3.4.21.37) was obtained from Athens Research & Technology (16-14-051200). Sterile preparations of α1PI, Prolastin-C (lot# 26NLK52) or Zemaira (lot# C405702), were generously provided by Grifols and CSL Behring, respectively, and were equivalent in activity.

AT-2 chemically inactivated simian/human immunodeficiency virus (SHIV 89.6) preparations, consisting of non-infectious virus with conformationally and functionally intact HIV envelope glycoproteins, were provided by the AIDS Vaccine Program (Leidos Biomedical Research, Inc., SAIC-Frederick, Frederick, MD).

The following representatives of the five classes of β-lactams were screened for activity:

I Cephalosporins (Cephems)
 1) Cephalexin (CAS# 15686-71-2, 347.4 mw) (Cayman Chemical 9002009, batch 0462553-11)
 2) Cefuroxime (CAS# 55268-75-2, 424.39 mw) (SigmaAldrich 34218-100 mg, batch SZBE050XV)
II Penicillins (Penams)
 1) D-Ampicillin (CAS# 63-53-4, 349.4 mw) (SigmaAldrich A9393-5 g, batch 086M4774V)
 2) Penicillin V (CAS# 87-08-1, 350.39 mw) (SigmaAldrich 1504489-200 mg, batch R05030)
 3) Dicloxacillin (CAS# 3116-76-5, 492.31 mw) (SigmaAldrich 46182-100 mg, batch SZBD263XV)
 4) Amoxicillin (CAS# 34642-77-8, 365.40 mw) (SigmaAldrich A8523-1G, batch 066M4760V)
 5) Alphataxin (CAS#19379-33-0, 349.4 mw) (BOC Sciences, batch B17LM02161)
III Monobactams
 1) Aztreonam (CAS# 78110-38-0, 435.43 mw) (SigmaAldrich A6848-50 mg, batch MKBW2997V)
 2) Ezetimibe (CAS# 163222-33-1, 409.43 mw) (SigmaAldrich 1269028-250 mg, batch F028D0)
IV Penems
 1) Faropenem (CAS# 122547-49-3, 307.30 mw) (SigmaAldrich F8182-10 mg, batch 0000016430)
V Carbapenems
 1) Doripenem (CAS# 148016-81-3, 420.50 mw) (SigmaAldrich 32138-25 mg, batch BCBR7602V)

**Antibiotic activity**. The Clinical and Laboratory Standards Institute (CLSI) approved protocol for the Kirby-Bauer Disk Test was performed to compare the antibiotic activities of D-ampicillin with Alphataxin (Turnidge and Paterson, 2007; Performance Standards for Antimicrobial Susceptibility Testing, 2014). D-Ampicillin-sensitive *E. coli* DH5-Alpha was cultured overnight in LB broth, and the cell concentration was calibrated using McFarland Turbidity Standard 0.05 for <300 × 10^6^ CFU. At this concentration of cells, bacteria were spread on Mueller-Hinton agar plates. Filter disks (6 mm diameter) were placed on the agar and to each disk was applied D-ampicillin (10 μl) or Alphataxin (10 μl) in 10-fold serial dilutions beginning with 50 mM concentration. After incubation for 16 h at 37°C, plates were examined for zones of inhibition.

**Inhibition of HLE-G enzymatic activity**. Two-fold serial dilutions of HLE-G in 100 µl Tris-buffered saline (pH 7.8) were added to rows of wells of a microtiter plate as previously described (Bristow et al., 1991). To columns of well were added a constant concentration of α1PI or β-lactams. The microtiter plate was incubated for 10 min at 37°C. The HLE-G substrate (Succinyl-L-Ala-L-Ala-L-Ala-p-nitroanilide, Sigma) was added to all wells, and absorbance at 405 nm was detected kinetically at 23°C using an MRX plate reader. The β-lactam concentration at which 50% HLE-G after 5 min incubation with substrate was determined to be the 50% inhibitory concentration (IC_50_).

**Cellular adherence**. Ten-fold serial dilutions of positive control α1PI or β-lactams at a beginning concentration of 100 nM and ending concentration of 0.01 nM in a volume of 10 μl were placed in wells of a 10-well microscope slide (Electron Microscopy Sciences). Human U937 Clone 10 cells or murine F12.23 cells (25 μl containing 5 × 10^3^ cells) were added to the wells and incubated for 30 min in humidified 5% CO_2_ at 37°C as previously described (Bristow et al., 2008). Unattached cells were removed by washing using AIM-V, and attached cells were fixed by application of 4% paraformaldehyde. Attached cells were counted by light microscopy.

**Receptor Polarization and Endocytosis**. To Eppendorf tubes that been precoated with RPMI-1640 containing 10% fetal bovine serum to prevent attachment, were added 250 μl containing 5 × 10^5^ U937 Clone 10 cells. Cells were conditioned with positive control α1PI, Alphataxin, or negative control AIM-V for 15 min at 37°C, 5% CO_2_ to induce polarization and aggregation of functionally-related plasma-membrane receptors including cell surface human leukocyte elastase (HLE-CS), CD4, CD184, T cell antigen receptor (TCR), and the very low density lipoprotein receptor (VLDLR) as previously shown (Bristow et al., 2013).

U937 Clone 10 cells were pulsed with SHIV particles (30 ng p27 or p24 per 10^6^ cells) for 2 h at 2°C, a circumstance that allows binding, but prevents endocytosis. Alternatively, cells were pulsed with virus for 2 h at 37°C, a circumstance that allows binding and endocytosis (Bristow et al., 2012). Following pulsing for 2 h, cells were pelleted, resuspended in AIM-V and allowed to adhere to Alcian blue coated microscope slides for 8 min at 4°C, washed, and fixed using 4% paraformaldehyde as previously described (Frank et al., 2002). The presence of AT-2 virus in test cells was detected using dodecameric human CD4-IgG_1_ provided by the Laboratory of Immunoregulation, NIAID, NIH. This reagent specifically recognizes conformationally intact HIV/SIV envelope gp120. CD4-IgG_1_ was detected using rabbit anti-human IgG conjugated with horse radish peroxidase (SigmaAldrich). CD4-IgG-labeled cells were coupled to Oregon 488 fluorochrome using the tyramide signal amplification system (Life Science Products, Boston, MA, United States). In some cases, cells stained on slides were permeabilized using 0.05% saponin during the blocking step and further stained with the nuclear staining dye, 4′,6-diamidino-2-phenylindole (DAPI), mounted, and examined by epifluorescence microscopy using a Zeiss Axioplan to detect positive and negative reactions. Representative samples of positive and negative reactions were subsequently captured by confocal microscopy using a Perkin Elmer Operetta High Content Imaging System. Cells were analyzed by confocal microscopy using 2 μm scanning from the attached surface toward the unattached surface. Confocal images of cells preconditioned with Alphataxin were captured 6 μm above the attached surface of the cells to determine uninternalized vs. internalized virus.

**Statistical Analysis**. Data were normally distributed unless stated otherwise. Means were compared using one-way analysis of variance or Student’s *t*-test. Apolipoproteins were not normally distributed, and medians were compared using one-way Kruskal-Wallis analysis of variance on ranks. Comparison of medians using the Mann-Whitney Rank Sum Test was used to determine statistical significance of differences between individual groups. Measurements are presented as the mean ± standard deviation. The power of test for all statistical analyses exceeded 0.8 suggesting the sample size was sufficiently large to obtain significance.

## Results

**FIGURE 1:**
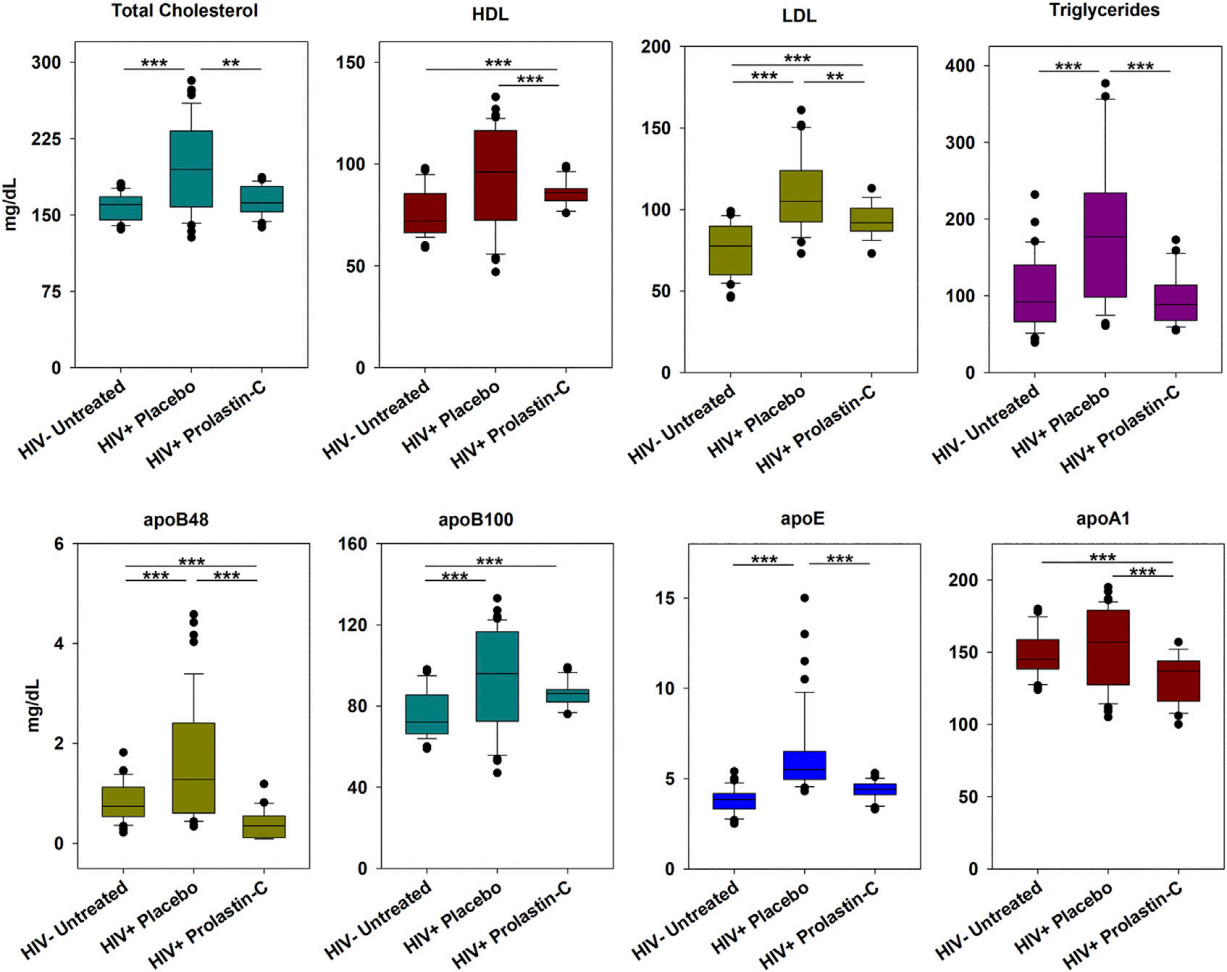
Prolastin-C treatment in HIV-1-infected individuals decreases circulating levels of lipoproteins other than apoB100. At baseline and each week for 8 weeks of treatment, individuals were assessed for lipoprotein levels. The clinical trial was composed of 3 arms: uninfected, uninfected (n = 4), HIV-1 infected, placebo-treated (n = 5), and HIV-1 infected, Prolastin-C treated (n = 3), and measurements for each arm contain n = 36, n = 45, and n = 27, respectively with the exception of LDL. One individual in the placebo arm and one in the Prolastin-C arm were omitted from analysis of LDL levels due to their treatment with LDL-lowering statin medications resulting in n = 36 and n = 18 measurements, respectively. Apolipoproteins were not normally distributed, and medians were compared using one-way Kruskal-Wallis analysis of variance on ranks and the Mann-Whitney Rank Sum Test to determine significance of differences between individual treatment groups. Medians are depicted within box plots. The power of test for all statistical analyses exceeded 0.8 suggesting that sample size was sufficiently large to obtain significance. Asterisks designate a statistically significant difference, **p < 0.01, ***p < 0.001.

**α1PI treatment of HIV-1-infected individuals decreases lipoproteins and apolipoproteins**. Because α1PI is in negative feedback regulation with LDL, we sought to examine the influence of α1PI treatment on lipoprotein and apolipoprotein levels. HIV-1-infected individuals, 89% of whom have below normal levels of α1PI were enrolled in a clinical trial and randomly assigned to receive 8 weekly treatments of α1PI (Prolastin-C) or placebo (Bristow et al., 2012). Untreated, uninfected individuals were enrolled for monitoring weekly changes. Clinical patients initiating Prolastin-C who were genetically deficient for α1PI (Pi-ZZ) were recruited to the study, but the study was not successful in enrolling volunteers. In addition to the standard lipoprotein panel (cholesterol, HDL, LDL, triglycerides), apolipoproteins apoB48, apoB100, apoE, and apoA1 were measured. HIV-1-infected individuals in the placebo arm exhibited greater than normal total cholesterol, triglycerides, LDL, apoB48, apoB100, and apoE levels, but not HDL or apoA1 levels **(** Figure 1 **)**. Following 8 weeks of weekly treatment with Prolastin-C, total cholesterol, triglycerides, and apoE levels decreased from above normal to normal levels, whereas HDL, apoB48, and apoA1 levels decreased from above normal to below normal levels. HIV-1-infected individuals exhibited greater than normal LDL and apoB100 levels, but after Prolastin-C treatment, greater than normal levels of B100 were maintained at the same levels as placebo treatment; in contrast, LDL levels in the Prolastin-C arm were significantly lower than in the placebo arm. Thus, Prolastin-C treatment of HIV-1-infected individuals decreased total cholesterol, HDL, LDL, triglycerides, apoB48, apoE, and apoA1, but did not significantly decrease apoB100 levels.

**β-Lactams bind to HLE-G and HLE-CS**. To screen β-Lactam compounds *in vitro* for the potential to act as surrogates for α1PI *in vivo*, compounds were initially examined for interaction with HLE-G, and the IC_50_ was determined. Based on the molar activity of HLE-G (75 μM), α1PI exhibited an IC_50_ of 38 µM which is within the margin of their known relatively equimolar relationship (Table 1, blue column). Cephems exhibited an average IC_50_ of 150.4 ± 23 μM, penams averaged 243 ± 36 μM, monobactams (including ezetimibe) averaged 255.4 ± 30 μM, a penem IC_50_ was 203 μM, and a carbapenem was 177.8 μM, suggesting that all five classes of β-lactams effectively bind to HLE-G at a 2–4 molar excess of β-lactams and that cephems exhibited the greatest affinity. Alphataxin, a penam, exhibited HLE-G interaction similar to cephems. These results suggested these β-Lactams might also interact with HLE-CS.

**TABLE 1:** All 5 Classes of β-Lactams Bind to HLE-G and HLE-CS.

Compound class	Compound	HLE-G		HLE-CS	
		IC_50_ (µM)^a^	Molar excess^b^	Adherent cells	Optimal concentration (nM)^c^
Cephems					
	Cephalexin	134.1	1.8	27 ± 10	10
	Cefuroxine	166.7	2.2	34 ± 8	1
Penams					
	D-Ampicillin	280.7	3.7	47 ± 8	1
	Pen V	262.0	3.5	30 ± 4	10
	Dicloxacillin	231.6	3.1	26 ± 9	1
	Amoxicillin	253.1	3.4	54 ± 20	1
	Alphataxin	187.7	2.5	39 ± 7	1
Monobactams					
	Aztreonam	234.1	3.1	33 ± 4	1
	Ezetimibe	276.7	3.7	40 ± 3	100
Penems					
	Faropenem	203.1	2.7	23 ± 4	10
Carbapenems					
	Doripenem	177.8	2.4	52 ± 6	100

a IC_50_ of compound vs. HLE-G (75 µM). For comparison, α1PI is 38 µM at IC_50_.

b Molar excess of compound to HLE-G at IC_50_.

c To each compound-treated well was added 2,000 U937 cells. For comparison, the optimal concentration of α1PI is 1 nM per 10,000 U937 cells yielding 75 ± 19 adherent cells.

To determine whether β-lactams interact with HLE-CS, we examined cellular adherence to glass as previously described (Bristow et al., 2008). The first step in cellular locomotion is adherence which is calcium independent and requires no nuclear signaling. Compounds were measured for the ability to induce human U937 Clone 10 cells and murine F12.23 cells to adhere to glass (Table 1, yellow column). The optimal adherence concentration for cephems and penams (1–10 nM) was comparable to α1PI (1 nM) and superior to monobactams, penems, and carbapenems (1–100 nM). Alphataxin, a penam, exhibited HLE-CS interaction similar to α1PI, cephems, and other penams suggesting it might be effective as a surrogate for α1PI *in vivo*.

**FIGURE 2:**
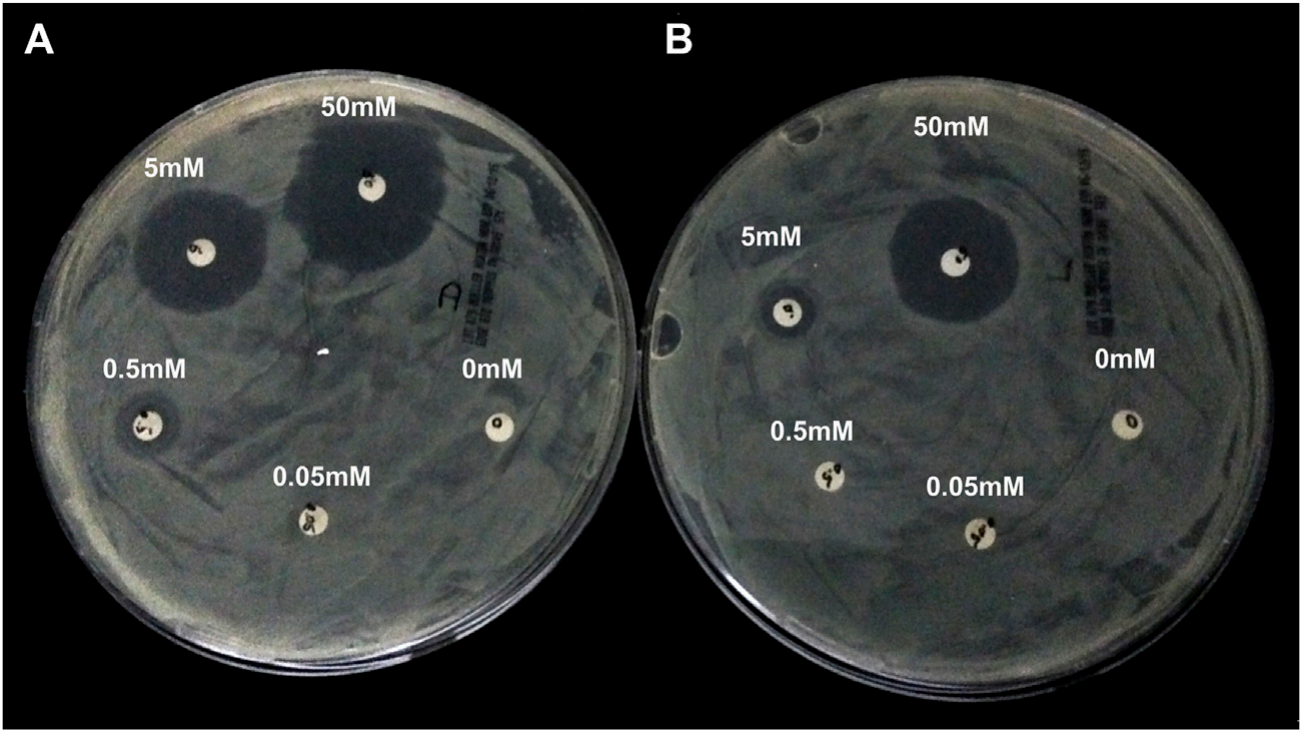
Lack of Antibiotic Activity by Alphataxin. D-Ampicillin-sensitive *E. coli* DH5-Alpha, calibrated using McFarland Turbidity Standard 0.05 for <300 × 10^6^ CFU, was spread on Mueller-Hinton agar plates. Filter disks (6 mm diameter) were placed on the agar and to each disk was applied D-ampicillin (10 μl) or Alphataxin (10 μl) in 10-fold serial dilutions, counterclockwise, beginning with 50 mM concentration. After incubation for 16 h at 37°C, plates were examined for zones of inhibition (clear agar). **(A)** The diameters of the zones of inhibition by D-ampicillin at concentrations of 50 mM (12 o’clock), 5 mM (10 o’clock), and 0.05 mM (8 o’clock) were 3.8, 3.0, and 1.4 cm, respectively. **(B)** The diameters of the zones of inhibition by Alphataxin at 50 mM (at 12 o’clock) and 5 mM (10 o’clock) were 2.8 and 1.5 cm, respectively. Images are representative of two repeated experiments.

**Alphataxin lacks effective antibiotic activity**. Because Alphataxin is an enantiomer of D-ampicillin, the bactericidal activity of Alphataxin was examined using the CLSI-approved protocol for measuring inhibition of growth of the D-ampicillin susceptible (S) bacteria *E. coli* DH5-Alpha in the presence of Alphataxin or D-ampicillin. According to the 2014 CLSI Table 2A, the minimal inhibitory concentration (MIC) of D-ampicillin is ≤8 µg/ml for inhibition of growth of *E. coli* (Performance Standards for Antimicrobial Susceptibility Testing, 2014). For *E. coli* that are categorized as resistant (R) to inhibition by D-ampicillin, MIC ≥32 µg/ml. The diameters of the zones of inhibition for D-ampicillin at concentrations of 0.5, 0.05, and 0.005 mM were 3.8, 3 and 1.4 cm, respectively (Figure 2A). The diameters of the zones of inhibition for Alphataxin at 0.5 and 0.05 mM were 2.8 and 1.5 cm, respectively (Figure 2B). We can conclude that the inhibitory dose of D-ampicillin in this assay was ≥0.005 mM (1.75 μg/ml), and that of Alphataxin was ≥0.05 mM (17.5 μg/ml). Because the minimum inhibitory dose of Alphataxin was 10-fold greater than the minimum inhibitory dose of D-ampicillin (≤8 µg/ml), the MIC is also 10-fold greater (≥80 µg/ml) which exceeds the Susceptibility Test Interpretive Criteria (STIC) or “breakpoint” reported in the 2014 CLSI Table 2A (≥32 µg/ml). Thus, Alphataxin is not an effective antibiotic against *E. coli*.

**FIGURE 3:**
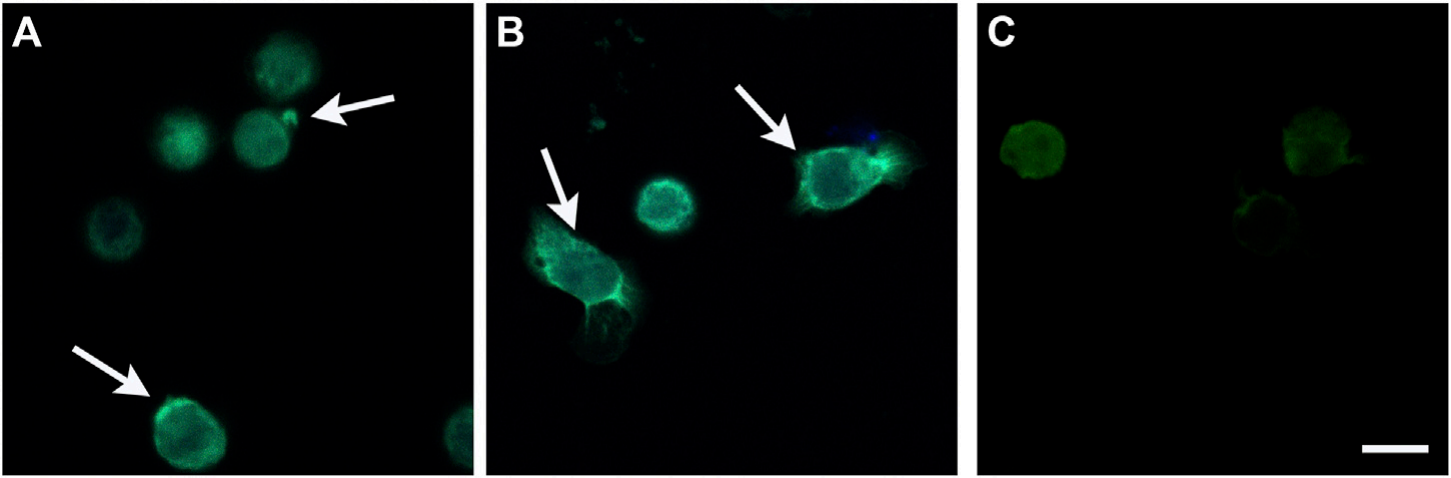
Stimulation of Cellular Locomotion and Endocytosis by Alphataxin. U937 Clone 10 cells were preconditioned with α1PI, Alphataxin, or buffer at 37°C for 15 min to induce polarization and receptor aggregation followed by incubation with SHIV at either 2°C or 37°C for 2 h. Cells were adhered to Alcian blue-coated slides and imaged by epifluorescence microscopy using a Zeiss Axioplan. Representative images were captured by confocal microscopy with a Perkin Elmer Operetta High Content Imaging System using 2 μm scanning from the attached surface toward the unattached surface. The three representative confocal images depicted were captured 6 μm above the attached surface of the cells. **(A)** Cells maintained at 2°C. Arrows denote receptor polarization. SHIV (bright green) was detected only on the exterior plasma membrane of polarized cells, never internal to the cells. **(B)** Cells maintained at 37°C. Arrows denote the leading edge and retracting trailing edge. SHIV (bright green) was detected internal to the cells at the leading edges (green tubes) and in endosomes (green dots) at the trailing edges of migrating cells. **(C)** Cells preconditioned with buffer alone and maintained at 2°C showing no SHIV binding. Bar represents 25 µm. Images are representative of 3 repeated experiments.

**Alphataxin induces cellular locomotion and endocytosis**. To screen whether Alphataxin mimics α1PI by inducing cellular locomotion, the localization of fluorescently-labeled receptor cargo (SHIV) was first screened by fluorescence microscopy to identify positive and negative reactions before capturing representative samples using confocal microscopy as previously described (Bristow et al., 2003). HIV-1 has been shown to infect cells via endocytosis, and endocytosis of CD4 and HIV-1-bound CD4 is induced when α1PI binds to HLE-CS on primary T cells or U937 Clone 10 cells (Moriuchi et al., 1998; Bristow et al., 2003; Hubner et al., 2009; Bristow et al., 2013). To examine whether Alphataxin similarly induces endocytosis of HIV-1-bound CD4, U937 Clone 10 cells were pretreated with Alphataxin to induce receptor polarization and subsequently incubated with SHIV. As detected by confocal microscopy, cells maintained at 2°C exhibited receptor polarization with SHIV detected only on the exterior plasma membrane of polarized cells, never internalized into the cells (Figure 3A). Cells were rounded and exhibited no evidence of cellular locomotion. In contrast, cells maintained at 37°C exhibited an extending leading edge and retracting trailing edge characteristic of cell migration (Figure 3B). SHIV was detected internal to the cells, prominently along tubular structures at the leading edges and in discrete endosomes at the trailing edges of migrating cells as previously disclosed (Bristow and Winston, 2021). No SHIV binding occurred in the presence of buffer alone (Figure 3C). Thus, Alphataxin mimics α1PI activity by binding to HLE-CS and inducing cellular locomotion and endocytosis.

**FIGURE 4:**
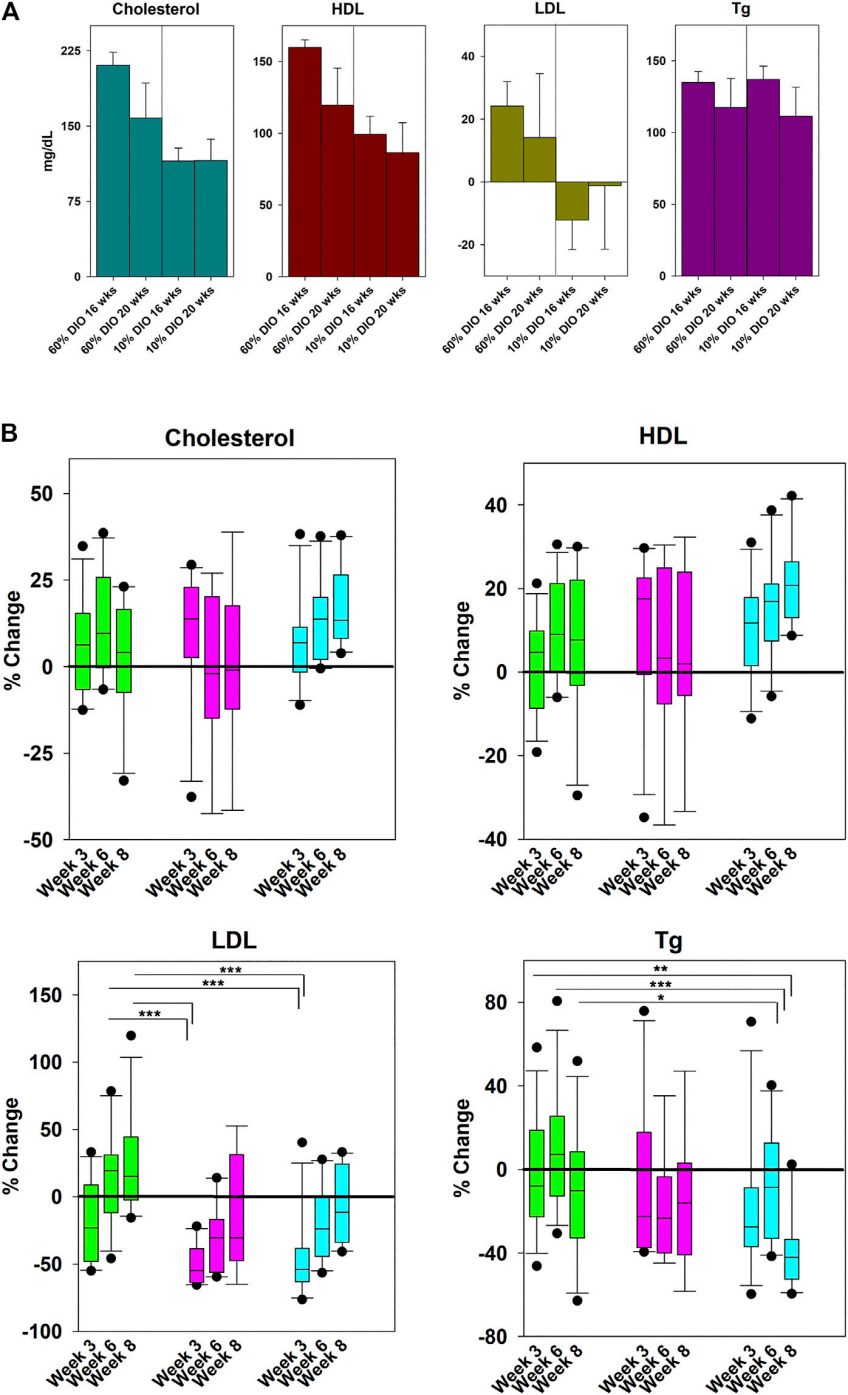
Effects of Diet and Drug Treatment on Cholesterol Levels. **(A)** To establish the effects of dietary fat on lipoprotein levels in the DIO mouse model, mice were fed a 60% or 10% fat diet (control) for 20 weeks. In mice fed a 60% fat diet, there were significantly higher total cholesterol levels (*p* < 0.001, n = 4 mice/diet), HDL levels (*p* < 0.001, n = 4 mice/diet), and LDL levels (*p* < 0.03, n = 4 mice/diet) than in mice fed a 10% fat diet. There was no difference between diets in triglyceride levels (*p* = 0.66, n = 4 mice/diet). **(B)** Mice on the 60% high-fat diet were treated daily by oral gavage with Zetia (ezetimibe, 10 mg/kg) or Alphataxin (5 mg/kg). To compare the effectiveness of ezetimibe (■) and Alphataxin (■) with vehicle (■), % change from baseline was calculated as [100-(Treatment-Baseline/Baseline*100)]. Data were not normally distributed. Treatment arms were compared one-way Kruskal-Wallis analysis of variance on ranks and the Mann-Whitney Rank Sum Test to determine significance of differences between individual treatment groups. Asterisks designate a statistically significant difference at the designated weeks, *p < 0.05, **p < 0.01, ***p < 0.001.

**Alphataxin lowers LDL levels**. Because LDL regulation is complex, it was desired to use a mouse model that was genetically intact, i.e., without a gene knockout, to examine whether Alphataxin lowers LDL levels. As an alternative, Alphataxin was tested using the well characterized DIO mouse model developed by Jackson Laboratory for studying their high fat diet on parameters of metabolic syndrome and diabetes. Before investigating whether Alphataxin might lower LDL levels, the DIO mouse model was first tested to confirm that high dietary fat content also caused changes in lipoprotein levels. For this purpose, DIO mice (C57BL/6J mice) were fed a defined diet that consisted of 10% fat (control) or 60% fat (high fat diet). After 16 weeks on the diets, in mice fed a 60% fat diet, as expected, mice exhibited significantly higher total cholesterol levels (*p* < 0.001, n = 4 mice/diet), HDL levels (*p* < 0.001, n = 4 mice/diet), and LDL levels (*p* < 0.03, n = 4 mice/diet) than in mice fed a 10% fat diet (Figure 4A). There was no difference between diets in triglyceride levels (*p* = 0.66, n = 4 mice/diet). These data show that the DIO mouse model provides an effective *in vivo* model of metabolic syndrome and diabetes for testing LDL-lowering drugs.

To determine whether Alphataxin lowers LDL levels, DIO mice on a 60% fat diet were treated daily by oral gavage with vehicle control, the penam Alphataxin, or the FDA-approved monobactam ezetimibe (Zetia). There were no significant changes in total cholesterol or HDL levels with any treatment (Figure 4B). LDL levels were significantly decreased after 3 weeks of treatment using ezetimibe (*p* < 0.001, n = 11) and Alphataxin (*p* < 0.001, n = 11) as compared with vehicle control (n = 12). Ezetimibe and Alphataxin were statistically comparable in effect (*p* = 0.90, n = 11, respectively). Thus, Alphataxin was equivalent to ezetimibe in lowering LDL levels. In contrast to ezetimibe, after 8 weeks of treatment, Alphataxin significantly lowered triglyceride levels as compared with vehicle control week 3 (*p* = 0.002, n = 10 and n = 12, respectively), week 6 (*p* < 0.001, n = 10 and n = 12, respectively, and week 8 (*p* = 0.03, n = 10 and n = 11, respectively).

## Discussion

Changes in lipoprotein and apolipoprotein levels following Prolastin-C treatment in HIV-1-infected individuals suggest that α1PI is not functionally related to apoB100 levels (endogenous lipoprotein transport pathway), but is directly or indirectly related to apoB48, apoE, apoA1, HDL, LDL, and triglyceride levels (endogenous and exogenous lipoprotein transport pathways. ApoA1 is found in chylomicrons and HDL; apoE is found in chylomicron remnants and HDL (Feingold, 2021). ApoB48, and apoA1 are produced in the intestine, as is α1PI (Xie et al., 1999). In addition, α1PI, apoA1, apoE, HDL, and LDL are produced in the liver. It is thought that apoB100, which incorporates in VLDL, is exclusively synthesized by liver hepatocytes and that apoB48, which incorporates in chylomicrons, is exclusively synthesized by intestinal enterocytes (Nakajima et al., 2014). The effects of Prolastin-C treatment on lipoprotein and apolipoprotein levels in subjects not infected with HIV-1 would be informative in future studies.

While α1PI and apoB100, but not apoB48, bind to LDL-RFMs, the evidence presented herein suggests that in addition to participating in the endogenous lipoprotein transport pathway (through the liver), α1PI may participate in the exogenous lipoprotein transport pathway (through intestinal lymph). We previously reported that α1PI and LDL are in negative feedback regulation and that HIV-1-infected individuals with below normal CD4^+^ T cells (< 500 cells/µl) show a striking linear correlation between LDL levels and active α1PI concentration (r^2^ = 0.92, *p* < 0.0001, n = 13) supporting evidence that α1PI participates in the exogenous lipoprotein transport pathway (Bristow et al., 2013). Chylomicrons from enterocytes are secreted into the lymphatics where they are transported through mesenteric lymph nodes to the thoracic duct and into blood (Dixon, 2010; Randolph and Miller, 2014; Hokkanen et al., 2019). The mechanism of chylomicron transport through lymph is not thought to be dependent of lymphocytes; however, the interaction of α1PI with LDL-RFMs to induce lymphocyte locomotion and the relationship between α1PI and apoB48 support the previously suggested, studies that lymphocytes participate in transport of dietary chylomicrons through mesenteric lymph nodes and the thoracic duct (Miura et al., 1993).

Considering the potential for α1PI to lower LDL levels whether by the exogenous or endogenous lipoprotein transport pathway, it was of interest to identify small molecules to act as surrogates for α1PI. Crystallographic studies of β-lactams binding to the active site of HLE-G at 1.84 Å was undertaken decades ago to determine whether β-lactams might be used to develop small molecule inhibitors of HLE-G (Navia et al., 1987). The crystallographic data demonstrate that β-lactams bind to the catalytic triad of HLE-G in the same manner as α1PI forming a covalent-like complex. We screened β-lactams for compounds that might similarly bind to HLE-G and HLE-CS. Because β-Lactams are antibiotics which have the capacity to promote the evolution of antibiotic resistant bacteria, we sought to modify β-lactams to eliminate their antibiotic activity.

β-lactam antibiotics are derived from prokaryotes which produce molecules with dextrorotation of incident polarized light whereas eukaryotes produce molecules that are levorotary. We developed enantiomers of β-Lactam structures that exhibit levorotation and demonstrate here that one such enantiomer, Alphataxin, lacks antibiotic activity and, not unexpectedly, has greater affinity for HLE-CS (a levorotary protein) than the dextrorotary β-Lactam enantiomer. Thus, Alphataxin, and other levorotary β-Lactams have the potential to be useful as small molecule surrogates of active or inactive α1PI.

There are five classes of β-lactams including cephems, penams, monobactams, penems, and carbapenems. Based on scarce *in vivo* evidence that antibiotic treatment using penams influenced the immune system in healthy, control animals, we chose a penam, Alphataxin, as our lead compound (Spurling et al., 1986; Tamura et al., 2013). In pre-clinical *in vitro* testing, Alphataxin was demonstrated here to have ineffective antibiotic activity well below the CLSI guidelines as compared with D-ampicillin, its dextrorotary enantiomer. β-lactams, including Alphataxin, bound to HLE-G within an order of magnitude of α1PI, 4-fold to 7-fold higher concentration than α1PI. β-lactams similarly bound to HLE-CS within the range of affinity exhibited by α1PI. The difference in α1PI affinity for soluble HLE-G and for plasma membrane-associated HLE-CS is attributed to the absence of water molecules and presence of lipid molecules proximate to HLE-CS (Bangalore and Travis, 1994). Alphataxin and α1PI exhibited equivalent optimal concentrations (1 nm) for binding to HLE-CS suggesting that Alphataxin might be suitable as an α1PI surrogate. In support of this conclusion, pre-clinical *in vivo* studies show here that Alphataxin effectively lowered LDL levels with equivalence to ezetimibe, a monobactam with FDA-approval for lowering LDL levels.

Considering that Alphataxin lowered triglyceride levels, yet ezetimibe did not, these two drugs appear to interact in lipoprotein regulation pathways with nuanced differences. Ezetimibe binds to the Niemann-Pick C1-like 1 protein (NPC1L1) and inhibits cholesterol absorption into enterocytes from the intestinal lumen (Burnett et al., 1994). We found here that ezetimibe also binds to HLE-CS as does Alphataxin. Yet due to the different effects of Alphataxin and ezetimibe on triglyceride levels, Alphataxin and ezetimibe appear to function through separate pathways even though they are both structurally β-lactams and both lower LDL levels. Interestingly, although ezetimibe is a β-lactam, its antibiotic effects are not readily apparent. It has been shown that ezetimibe influences the intestinal microbiome by increasing the *Lactobacillus spp.* and to kill Leishmania amazonensis promastigotes with a nIC_50_ of 30 μM (Catry et al., 2015; Andrade-Neto et al., 2016). Additional studies are needed to determine the mechanisms that discriminate Alphataxin and ezetimibe effects, for example, whether HLE-CS, an esterase, is involved in cholesterol absorption by enterocytes, whether Alphataxin binds to NPC1L1, and whether ezetimibe has antibacterial activity.

At the time that ezetimibe was discovered to lower LDL levels, there was no known mechanism to explain its activity and Schering-Plough Research Inst. undertook to discover the underlying mechanism for the purpose of obtaining FDA approval (Clader, 2004). Despite the lack of mechanistic explanation for Alphataxin to lower apoB48 levels, the mechanism for lowering LDL levels is by acting as a surrogate for α1PI. While currently available therapeutics are effective, all drugs have serious adverse effects to the point that many of those who need LDL-lowering drugs may be unable to use them. There is a striking need for new therapeutics for lowering LDL levels to prevent cardiovascular disease, a disease that affects 30% of the global population. The preclinical data presented here suggest that β-lactams, with a long history of safety and lack of muscle pain, provide promising, effective therapeutics for lowering LDL levels when in their levorotary conformation.

## Data Availability

The original contributions presented in the study are included in the article/Supplementary Material, further inquiries can be directed to the corresponding author.
